# 肺腺癌化疗联合PD-1单抗免疫治疗继发重度贫血病例报道及文献复习

**DOI:** 10.3779/j.issn.1009-3419.2025.102.25

**Published:** 2025-06-30

**Authors:** Yaowen HU, Jing ZHAO, Xiaoxing GAO, Yan XU, Mengzhao WANG

**Affiliations:** ^1^100730 北京，中国医学科学院，北京协和医学院，北京协和医院消化内科（胡耀文）; ^1^Department of Gastroenterology; ^2^呼吸与危重症医学科（赵静，高晓星，徐燕，王孟昭）; ^2^Department of Pulmonary and Critical Care Medicine, Peking Union Medical College Hospital, Chinese Academy of Medical Sciences and Peking Union Medical College, Beijing 100730, China

**Keywords:** 肺肿瘤, 免疫检查点抑制剂, 程序性细胞死亡受体1, 免疫相关不良事件, 贫血, Lung neoplasms, Immune checkpoint inhibitors, Programmed cell death 1, Immune-related adverse events, Anemia

## Abstract

程序性细胞死亡受体1（programmed cell death 1, PD-1）单抗治疗肺腺癌可能引起罕见而严重的血液学不良反应，可出现重症贫血等表现。尽管糖皮质激素被推荐用于免疫相关不良事件的管理，但针对PD-1单抗诱发的重症贫血的治疗经验仍十分有限，其疗效和安全性尚未充分验证。本文报道了1例化疗联合PD-1单抗治疗后发生重症贫血的晚期肺腺癌患者，经过系列检查，考虑诊断炎症性贫血，经足量糖皮质激素治疗后，血红蛋白显著回升，以期为临床中这类罕见血液学毒性的识别和治疗提供新的见解。

## Abstract (en)

Programmed cell death 1 (PD-1) inhibitor therapy for lung adenocarcinoma may induce rare but severe hematologic adverse events, including severe anemia. Although glucocorticoids are recommended for managing immune-related adverse events, therapeutic experience with PD-1 inhibitor-induced severe anemia remains limited, and its efficacy and safety have not been fully validated. This article reports a case of advanced lung adenocarcinoma in which severe anemia developed following combination therapy with chemotherapy and PD-1 inhibitor. After comprehensive evaluation, the patient was diagnosed with anemia of inflammation (AI) and achieved significant hemoglobin recovery following high-dose glucocorticoid treatment. These findings may provide new insights into the recognition and management of this rare hematologic toxicity in clinical practice.

原发性支气管肺癌已成为我国恶性肿瘤死亡的主要原因^[1]^。近年来，对于无法手术切除的患者，免疫治疗成为非小细胞肺癌患者的主要治疗策略之一^[2]^。驱动基因阴性的晚期肺腺癌患者，可选择程序性细胞死亡受体1（programmed cell death 1, PD-1）或程序性细胞死亡配体1（programmed cell death ligand 1, PD-L1）抑制剂行免疫治疗，尤其对于PD-L1高表达（≥50%）的患者临床获益更为显著^[3]^。然而，免疫治疗也伴随着一系列免疫相关不良事件（immune-related adverse events, irAEs），研究表明，化疗联合免疫治疗后贫血的发生率较高，多为轻度贫血，其主要原因可能与化疗对骨髓造血功能的抑制^[4]^有关，少许情况下可出现免疫治疗相关不良反应，包括溶血性贫血或者再生障碍性贫血等^[5]^。本文报道1例肺腺癌患者在接受化疗联合PD-1单抗免疫治疗后继发重度贫血，经过系列检查提示炎症性贫血（anemia of inf lammation, AI）的病例，旨在探讨其临床管理策略。

## 1 病例资料

患者男，75岁，因“发现右肺上叶占位4月，诊断肺癌3月余”就诊。2023年6月15日患者因皮疹外院查胸部计算机断层扫描（computed tomography, CT）发现右肺上叶前段肿块影（5.5 cm×7.6 cm），纵隔及肺门区淋巴结肿大，右肺上叶多发实性结节。全身正电子发射计算机断层显像（positron emission tomography/CT, PET/CT）提示右肺上叶前段肿块，约6.9 cm×4.9 cm，中央为放射缺失区，周边实性部分放射性摄取异常增高，最大摄取值（maximum standardized uptake value, SUVmax）12.2，考虑恶性病变可能性大，伴中央坏死；右肺上叶前段多个放射性摄取异常增高的实性结节，SUVmax 8.1，不除外转移；右侧肺门、纵隔淋巴结放射性摄取异常增高，SUVmax 9.0，考虑转移可能性大；头部磁共振成像（magnetic resonance imaging, MRI）及骨扫描未见明确转移。肺穿刺活检病理确诊为肺腺癌，免疫组化示细胞角蛋白7（cytokeratin 7, CK7）（+），P40（-），甲状腺转录因子1（thyroid transcription factor 1, TTF-1）（+），间变性淋巴瘤激酶（anaplastic lymphoma kinase, ALK）-D5F3（-），PD-L1肿瘤阳性细胞比例分数（tumor proportion score, TPS）为0%，因组织标本不足，无法行基因检测，期间检查示血常规提示血红蛋白（hemoglobin, Hb）为94 g/L。2023年8月5日患者行第1程化疗联合免疫治疗，具体为：培美曲塞800 mg d1+卡铂500 mg d1+帕博利珠单抗200 mg d1。2023年8月15日复查血常规提示：白细胞（white blood cell, WBC）为2.72×10^9^/L，Hb为98 g/L，血小板（blood platelet, PLT）为121×10^9^/L，给予人粒细胞集落刺激因子（granulocyte colony stimulating factor, G-CSF）治疗后复查血象恢复正常。因骨髓抑制，2023年8月25日行第2程减量治疗，具体为：培美曲塞700 mg d1+卡铂300 mg d1+帕博利珠单抗200 mg d1。第2程治疗后患者出现乏力、心悸、头晕，2023年9月5日复查：WBC为1.4×10^9^/L，中性粒细胞（neutrophil, NEUT）为0.48×10^9^/L，Hb为72 g/L，PLT为87×10^9^/L，予G-CSF治疗，2023年9月11日复查：Hb为52 g/L，粒系、PLT恢复正常；血肌酐为85 μmol/L，超敏C反应蛋白（hypersensitive C-reactive protein, hs-CRP）为105.00 mg/L（参考范围0-8 mg/L）。急诊就诊，输注2 U红细胞支持治疗，2023年9月28日复查血常规示Hb为59 g/L，再次输注2 U红细胞。2023年10月8日患者出现面部及四肢浮肿、胸闷，卧位憋气加重，否认胸痛，查体提示皮肤苍白，眼睑水肿，睑结膜苍白，双肺呼吸音粗，右上肺呼吸音低，双下肺散在湿啰音，心脏及腹部查体无殊，颜面部及四肢可凹性水肿；复查Hb为65 g/L，hs-CRP为277.40 mg/L，N末段B型钠尿肽原为11,006 pg/mL（参考范围0-450 pg/mL），高敏心肌肌钙蛋白I（hypersensitive cardiac troponin I, hscTnI）为88 ng/L（参考范围≤54 ng/L），心电图提示心率112次/min，完全右束支传导阻滞，考虑急性心功能不全。2023年10月17日收入北京协和医院呼吸与危重症医学科，10月18日完善血常规：WBC为9.59×10^9^/L，NEUT为8.40×10^9^/L，Hb为57 g/L，PLT为133×10^9^/L；网织红细胞（reticulocyte, RET）为131.00×10^9^/L（参考范围24.0-84.0×10^9^/L），外周血细胞形态学分析：红细胞大小不等，部分形态不规则，中心淡染区扩大；肝肾功能：丙氨酸氨基转移酶为25 U/L，碱性磷酸酶为230 U/L，血肌酐为82 μmol/L；hs-CRP为222.60 mg/L，类风湿因子（rheumatoid factor, RF）处于正常范围，白介素-6（interleukin-6, IL-6）为212.31 pg/mL，IL-8为121.43 pg/mL，铁蛋白（ferritin, Fer）为7524 ng/mL（参考范围24-336 ng/mL），转铁蛋白饱和度（transferrin saturation, SAT）为21.3%，维生素B12大于1500 pg/mL。促红细胞生成素（erythropoietin, EPO）为25.86 mIU/mL（参考范围4.5-31.88 mIU/mL）。血浆游离血红蛋白（plasma free hemoglobin, FHb）为3.4 mg/dL（参考范围0-5.0 mg/dL），尿胆原、尿胆红素阴性。直接抗球蛋白试验（IgG/C3d）阴性。尿潜血阴性，粪便外观为黄色成型便，便潜血阳性共3次。完善痰细菌真菌涂片及培养，未见明确致病菌，外周血需氧及厌氧培养阴性共1套。心肌损伤标志物：B型钠尿肽（brain natriuretic peptide, BNP）为240 ng/L，肌红蛋白为292 μg/L，hscTnI为82 ng/L。10月18日完善骨髓细胞形态检查：增生活跃，红系各阶段形态及比例大致正常，未见异常细胞；骨髓活检：（髂后上棘）骨髓组织中造血组织比例略减少，红系比例减少为著（图1）。10月19日完善胸部CT，见原发灶体积显著缩小（图2）；完善冠脉CT血管造影（CT angiography, CTA）：未见冠脉中重度狭窄。患者拒绝完善内镜检查。

**图 1:**
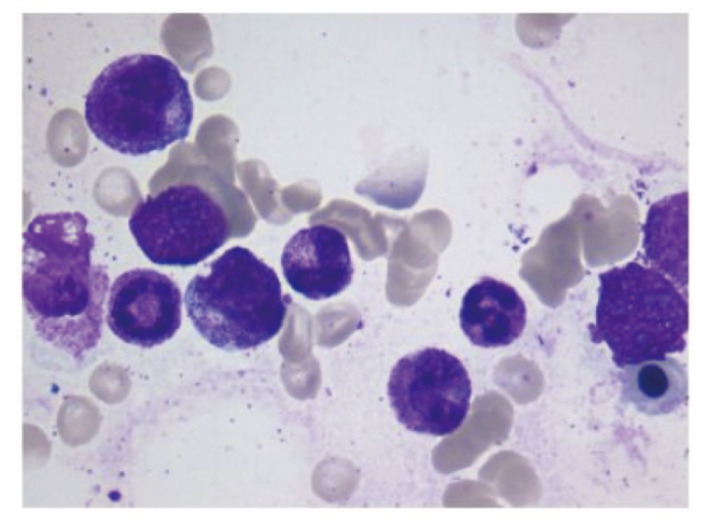
骨髓细胞形态学检查：增生活跃伴红系各阶段细胞形态正常（×100）

**图 2:**
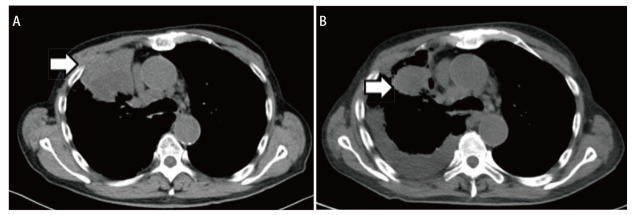
肺腺癌患者免疫联合化疗治疗前后胸部CT对比：原发灶体积显著缩小。 A：治疗前胸部CT纵隔窗；B：免疫联合化疗治疗2个周期后胸部CT纵隔窗。

结合患者辅助检查结果，血液科会诊考虑符合AI，前期化疗的骨髓抑制也部分参与其中。10月18日、10月20日共予4 U红细胞对症输血治疗，10月20日加用甲强龙40 mg qd静脉输注抗炎治疗，7 d后减量至泼尼松30 mg qd口服，复方磺胺甲恶唑片预防感染治疗；10月20日至10月24日予EPO 1万单位qod皮下注射治疗共3次；艾司奥美拉唑口服抑酸治疗，预防消化道出血。加用甲强龙治疗后，监测Hb由57 g/L回升并稳定于98-104 g/L，hs-CRP由277 mg/L降至42 mg/L。心脏方面，入室后予美托洛尔抗心室重构治疗，呋塞米利尿治疗，监测BNP由240 ng/L降至82 ng/L，hscTnI降至35 ng/L，下肢水肿完全缓解。予出院，出院5 d后复查Hg仍可稳定于105 g/L。

## 2 讨论

本例肺腺癌患者接受化疗联合帕博利珠单抗免疫治疗2个周期，出现Hb持续下降至重度贫血，合并心功能不全。贫血病因分析方面，需首先排查化疗相关骨髓抑制参与。患者在使用帕博利珠单抗同时还应用卡铂和培美曲塞，培美曲塞是叶酸代谢途径抑制剂，用药后可能出现严重血液系统毒性，如PLT减少、WBC减少，也有导致反复贫血^[6-8]^；铂类药物也可能导致严重的骨髓抑制^[9]^。患者化疗后出现一过性粒细胞缺乏、PLT减少，但本例患者贫血呈进行性恶化，其余两系均已恢复，不符合化疗骨髓抑制的典型表现；显著升高的hs-CRP、IL-6及铁代谢异常（高铁蛋白、正常SAT）均指向全身炎症反应，在糖皮质激素治疗后Hb快速回升，炎症指标下降，可符合免疫相关性AI的预期疗效，不支持化疗后骨髓抑制所致贫血。其他免疫相关贫血原因方面，患者直接抗球蛋白试验（包括IgG及C3d）阴性，FHb正常范围，可排除自身免疫性溶血性贫血及血管内溶血性贫血；骨髓造血组织比例大致正常，RET计数升高，可排除再生障碍性贫血；患者RF正常范围，无关节疼痛症状加重，不支持类风湿关节炎活动所致贫血；尽管粪便潜血阳性，但患者活动性消化道出血证据不足，失血所致贫血可能性小。该患者患有肺腺癌，免疫治疗后出现Hb进行性下降，辅助检查提示hs-CRP及IL-6显著升高，铁利用障碍，骨髓红系造血组织比例减低。根据AI的诊断标准：轻、中度的正细胞正色素性贫血；存在系统性炎症（如CRP和红细胞沉降率增高）；限制性铁利用（血清铁减少，SAT>15%，血清铁蛋白>12 μg/L）^[10,11]^，患者可符合AI。停用帕博利珠单抗并予足量糖皮质激素治疗后Hb水平显著回升并维持稳定，炎症指标水平下降。药品不良反应诺氏评分量表评分为7分，提示AI很可能与免疫治疗药物帕博利珠单抗有关。

帕博利珠单抗为PD-1抑制剂，其常见的irAEs主要为皮肤、消化和呼吸系统损伤^[12]^，血液免疫相关不良事件（haematological irAEs, hem-irAEs）的发生率为0.04%-3.6%，死亡率为14%，其中Hb相关irAEs发生率为0.17%-0.5%^[5,13]^。研究^[13]^表明抗PD-1或抗PD-L1会导致多种贫血类型，包括自身免疫性溶血性贫血、再生障碍性贫血等。目前抗PD-1治疗的血液学毒性机制尚未完全阐明。现有研究^[13]^表明，hem-irAEs相关自身免疫性溶血性贫血既有温抗体类型，也有冷抗体类型，这表明了抗PD-1或抗PD-L1免疫疗法所导致的免疫毒性的多样性。

尚无研究阐明免疫检测点抑制剂（immune checkpoint inhibitors, ICIs）导致AI的病例及确切机制。有研究^[15]^表明，ICIs诱导的T细胞活化通常会引发炎症或自身免疫类副作用，即irAEs^[14]^。在irAEs的发展过程中，不仅Th1和Th17等细胞的反应增强，炎症细胞因子水平如IL-6、IL-17、肿瘤坏死因子α（tumor necrosis factor α, TNFα）和IL-1β也随之升高。另有研究^[16]^表明ICIs治疗后IL-6、IL-17、趋化因子CXC配体9（C-X-C motif chemokine ligand 9, CXCL9）和CXCL10的水平显著上升，并伴随irAEs的发生。与之相对，IL-17水平的下降被观察到与症状缓解相关^[17]^；IL-17A抑制剂可有效治疗皮肤相关irAEs^[18]^；在一项纳入92例患者的研究^[19]^中，73%的irAEs在抗IL-6R治疗后缓解至≤1级。考虑到AI的根本原因为炎症因子（如IL-6）的释放，诱导下游的铁调素水平异常升高，导致功能性铁缺乏，限制红细胞生成；同时，炎症因子抑制EPO的产生和作用，进一步抑制红细胞生成^[10]^。PD-1抑制剂通过抑制T细胞表面PD-1抗原，可能导致T细胞过度激活，造成全身炎症反应，进而导致AI。

治疗方面，对于hem-irAEs，现有证据支持使用皮质类固醇进行初始治疗和支持治疗，包括根据需要输血^[5]^。本例患者经足量激素、抗感染及输血支持治疗后，Hb回升且维持稳定，炎症指标下降，提示治疗有效。

本文报道了1例帕博利珠单抗在晚期肺腺癌中的罕见的血液学不良反应。结合患者实验室检查及治疗反应，综合判断为AI，可能与免疫治疗诱发全身炎症反应相关。通过糖皮质激素治疗及密切监测，患者的贫血得到有效改善，为类似不良反应的处理方法提供了支持。
